# Effect of improving food security on parenting practices and caregiver–adolescent relationships: qualitative findings of an income-generating agricultural intervention in rural Kenya

**DOI:** 10.1192/bjo.2024.802

**Published:** 2024-12-26

**Authors:** Maricianah A. Onono, Lila Sheira, Edward A. Frongilio, Gladys Odhiambo, Pauline Wekesa, Amy Conroy, Elizabeth A. Bukusi, Craig R. Cohen, Sheri D. Weiser

**Affiliations:** Centre for Microbiology Research, Kenya Medical Research Institute, Nairobi, Kenya; Division of HIV/AIDS and Center for AIDS Prevention Studies, Department of Medicine, University of California San Francisco, CA, USA; Department of Health Promotion, Education, and Behavior, University of South Carolina, South Carolina, SC, USA; Division of Prevention Science, Center for AIDS Prevention Studies, University of California San Francisco, CA, USA; Department of Obstetrics, Gynecology & Reproductive Sciences, University of California San Francisco, CA, USA; Department of Medicine, Division of HIV, Infectious Diseases and Global Medicine and Center for AIDS Prevention Studies, University of California San Francisco, CA, USA

**Keywords:** Parenting stress, adolescent psychosocial well-being, structural interventions, food insecurity, adolescent behavioural

## Abstract

**Background:**

Despite the recognised links between food insecurity and parenting, few studies have evaluated the perceived impacts of livelihood or food security interventions on parental practices, intra-household functioning, adolescent behaviour and psychosocial outcomes in HIV-affected households in sub-Saharan Africa.

**Aims:**

The study aimed to understand the perceived effects of food security on parenting practices and how this was experienced by both adolescent girls (aged 13–19 years) and their caregivers in rural Kenya.

**Method:**

We conducted semi-structured, individual interviews with 62 caregiver–adolescent dyads who were participants in the adolescent *Shamba Maisha* (NCT03741634), a sub-study of adolescent girls and caregivers with a household member participating in the *Shamba Maisha* agricultural and finance intervention trial (NCT01548599). Data were analysed following the principles of thematic analysis.

**Results:**

Compared to control households, the *Shamba Maisha* intervention households had improved food security and strengthened economic security, which, in turn, improved parenting practices. Intervention households described changes in parenting experiences, including decreased parental stress, reduced absenteeism and harsh parenting and improved caregiver– adolescent relationships. These positive caregiving practices, in turn, contributed to improved mental health and fewer behavioural problems among adolescent girls. Changes in the control households were less noticeable.

**Conclusion:**

These findings demonstrate how an income-generating agricultural intervention may improve food security and positively affect parenting practices, intra-household dynamics and adolescent psychosocial well-being and behaviour. Further research is needed to explore how to harness the social benefits of agricultural interventions to best address the critical intersections among food insecurity, parenting practices and adolescent mental health.

One in four households in sub-Saharan Africa are classified as severely food insecure, and nearly one in every two are likely to be deficient in micronutrients during the dry season.^1,2^ Households are food insecure if they have ‘limited or uncertain availability of nutritionally adequate and safe foods or limited or uncertain ability to acquire acceptable foods in socially acceptable ways’.^3^ The experience of food insecurity has other harmful consequences for the well-being of adults, adolescents and children beyond malnutrition. Adolescents often bear a greater burden of household food insecurity compared to younger children. In contrast to younger children who may be prioritised through household-level assistance or programmes like school feeding, adolescents may not have access to these programmes or receive priority as they are viewed as being more self-reliant and consequently receive less attention.^4^ Previous studies among adolescents have found a link between food insecurity and increased developmental risk, including sexual risk-taking, as well as psychosocial disorders such as anxiety and depression.^5,6^ Adolescent girls are more likely to report food insecurity than boys and experience more significant effects than their male counterparts.^7^ Adolescent young women and girls are often responsible for preparing and serving food but often eat last and least, and in cases of food insufficiency, they eat less nutritious food or fail to eat altogether.^8^ In addition to the direct effects of food insecurity on adolescents, food insecurity and related economic stressors may also affect the quality of parenting and parental psychological functioning. Compromised parenting can negatively affect adolescent psychosocial well-being and behaviour.^9^

## Impact of economic and food insecurity on parenting and adolescent well-being

The parenting role demands that a parent meets food, educational and healthcare needs for all their children, including adolescents.^10^ When family economic pressure (such as food insecurity) is high, parents are at an increased risk for emotional distress (parental stress). This distress may, in turn, lead to disrupted family relationships, marital conflict and harmful caregiving practices, thereby affecting adolescent psychosocial adjustment.^11^ Food and economic insecurity can lead to less time for interaction between parents and their adolescents because of long working hours to obtain resources to fulfil caregiving roles. This prolonged absenteeism reduces the caregiver–adolescent connection and the parent's ability to guide the behaviour of their adolescents.^12^ HIV-affected families experience increased food insecurity, poverty,^13^ parental depression^14^ and a general lack of social support.^15^ Since these factors are also associated with reduced positive parenting, they may exacerbate negative behavioural and psychosocial outcomes among adolescent girls living in these HIV-affected households. Studies show that food insecurity is directly associated with parental connection, orientation towards success and adolescent uncertainty about the future.^16^

## Research gaps in food insecurity interventions and caregiver–adolescent relationships

Although the relationship between food insecurity and parenting practices is becoming increasingly well established, many prior studies have focused primarily on the effect of food insecurity on parental feeding practices and food consumption in high-income countries.^17^ The concentration of severe food insecurity in low-resource compared to high-resource countries, combined with the differences in social safety net arrangements, creates an urgent need for more research from low- and middle-income countries.^18^ There is also limited data on whether food insecurity interventions improve parental practices, specifically whether intervening at the household level to improve food security could improve the caregiver–adolescent relationship. To address the gap in the literature on linkages between food insecurity interventions and caregiver–adolescent relationships, we undertook a qualitative study among caregiver–adolescent dyads participating in the *Shamba Maisha* intervention. Our goal was to explore the perceived impact of changes in food insecurity through an income-generating agricultural intervention on intra-household dynamics, parenting practice, caregiver–adolescent relationships and the downstream psychosocial and behavioural well-being of adolescent girls in HIV-affected households in south-western Kenya.

## Method

This was a sub-study of the *Shamba Maisha* project, which wa*s* a matched-pair cluster randomised controlled trial (RCT) of a multi-sectoral agricultural and finance intervention among HIV-infected farmers on antiretroviral therapy (ART) to improve household food security and HIV health (NCT01548599).^19^ The main study was conducted by researchers from the University of California San Francisco (UCSF) and Kenya Medical Research Institute. This qualitative sub-study using semi-structured in-depth interviews (IDIs) with caregiver–adolescent pairs was conducted concurrently with the main study between April and December 2019 to qualitatively assess the perceived impact of food security on parenting practices and caregiver–adolescent relationships. This paper was written following the Consolidated Criteria for Reporting Qualitative Research (COREQ) guidelines to ensure comprehensive and transparent reporting of the qualitative research conducted (Supplementary file 1 available at https://doi.org/10.1192/bjo.2024.802).

### Setting

**Fig. 1:**
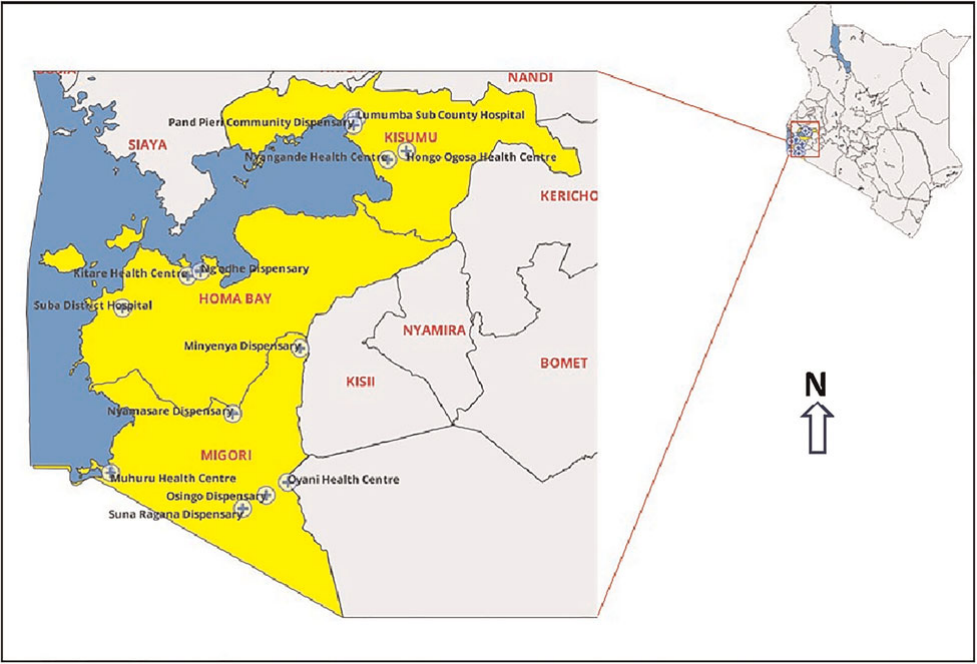
Map of the study region and sites.

Homabay, Kisumu and Migori Counties in south-western Kenya, where this study was conducted (see Fig. 1), have high rates of both HIV and food insecurity.^20^ At the end of 2018, prevalence rates of HIV were 19.6%, 17.5% and 13% in Homabay, Kisumu and Migori, respectively, making up nearly half of all HIV cases nationwide. Furthermore, in this region, adolescent girls and young women are nearly four times as likely as their male counterparts to be infected (11% *v.* 3%).^20^ The region also has the highest teenage pregnancy rate (27%) and 40% of households report lacking food or money to purchase food.^21^

### *Shamba Maisha* intervention

A comprehensive description of the *Shamba Maisha* intervention has been previously published.^22^ Briefly, the intervention was a multi-sectoral intervention aimed at addressing causes of food insecurity and poor health outcomes partly occasioned by a lack of irrigation in areas of unpredictable rainfall patterns and increasing drought frequency owing to climate change. The intervention consisted of the following: (a) a market interest loan of US$175 to purchase agricultural inputs (seeds, fertilisers and pesticides); (b) a human-powered water pump provided on loan; and (c) education in financial management and sustainable and regenerative farming practices.

### Recruitment and consenting

Between 24 April 2019 and 20 December 2019, caregiver–adolescent dyads were recruited from the intervention (*n* = 38) and control (*n* = 24) arms of the *Shamba Maisha* study (*N* = 62). The difference between the numbers in the two arms (38 *v.* 24) was based on theoretical saturation, where we reached saturation point earlier in the control compared to the intervention. The caregiver–adolescent dyads were purposively recruited during the end of their participation in the main study. Our purposive sampling strategy was guided by the following variables: among caregivers we considered the gender, age, education, marital status, household size and study arm. Among adolescents we considered the age, education level and study arm. Table 1 provides baseline characteristics of the caregiver–adolescent dyads. We defined primary caregivers as individuals primarily responsible for preparing food and seeking healthcare for adolescents (13–19 years) when needed. We excluded adolescents if they were (a) living with HIV at the start of the intervention period, (b) married, (c) heads of their households, (d) had inadequate cognitive and/or hearing capacity or (e) did not speak Dholuo, Swahili or English. Participants received information about the study and provided written consent if they were aged 18 + or assent if aged 13–17. Parents/guardians who participated in the study and had adolescents aged 13–17 years provided their own informed consent and that of their adolescents. The study focused on adolescent girls and young women because they tend to be more prone to challenges of food insecurity than boys.

**Table 1:** Baseline characteristics of the caregiver–adolescent dyads

Attribute	Frequency	Percent
Gender of caregiver		
Male	9	29
Female	22	71
Age of caregiver		
<40	10	32
≥40	21	68
Education level of caregiver		
Primary	22	71
Post-primary	9	29
Marital status of caregiver		
Married	20	69
Widowed	9	31
Household size of caregiver		
2–6	13	42
>6	18	58
Age of adolescent		
13–16	16	59
17–19	11	41
Education level of adolescent		
Primary	13	48
Post-primary	14	52
Study arm		
Intervention	19	61
Control	12	39

### Study's theory of change

**Fig. 2:**
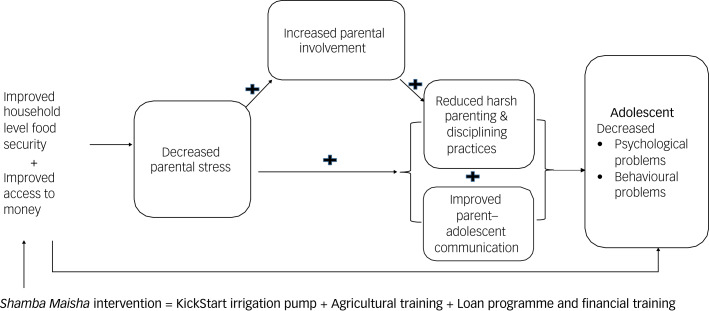
Links between household food security and parenting experience.

Figure 2 represents our theory of change for changes in adolescent health in areas with a high poverty level, food insecurity, significant mental health challenges and high HIV risk behaviour. Our theory of change indicates that enhancing household food security and improving cash flow among caregivers of adolescent girls results in decreased parental stress, which in turn increases parental availability and involvement in adolescent issues. In addition, decreased parental stress and increased parental involvement result in reduced harsh parenting and disciplining and improved caregiver–adolescent communication, giving rise to healthy adolescents with decreased psychological and behavioural challenges.

### Data collection

The semi-structured interview guides (Supplementary files 2 and 3) were developed in English, translated into Dholuo and Kiswahili, and then back-translated into English to check for translation accuracy. The guides were piloted among representatives of the target population to ensure that the questions were clear and that they elicited the desired information. Two trained female trilingual (English, Dholuo and Kiswahili) qualitative interviewers underwent rigorous training to conduct IDIs with adolescents and caregivers. Both interviewers held degrees in social science and public health. This training encompassed techniques for establishing rapport, active listening, asking open-ended questions and maintaining a non-judgemental and empathetic demeanour. Consenting/assenting participants were interviewed for approximately 60–90 min in a language of their choice. The qualitative interviewers were not in any way involved in the implementation of the main study. Topics covered in the IDIs included family composition and setup, dynamics within the family, family economic situation, household food security, caregiver–adolescent communication and relationships, barriers and facilitators to positive communication/parenting, mental health, adolescents’ sexual behaviour and experiences with and perceived impacts of the *Shamba Maisha* intervention on the above domains. The interviews for caregivers and adolescents were conducted separately in private spaces, and participants were reassured of their confidentiality. All the interviews were audio-recorded.

### Data analysis

Audio files were promptly transcribed verbatim and translated into English, and every 15th transcript was picked and checked for completeness and accuracy. All transcripts passing the check were redacted of any personally identifying participant information and uploaded for coding. A rigorous process alternating among interview transcripts, codes, discussion notes, field notes and relevant empirical and theoretical literature on the topic led to the final themes. Particular attention was paid to documenting how latent or interpreted meanings were derived from text, considering translation and context issues in this study. The primary interviewer coded all transcripts using Dedoose version 9.2.22 for MacOS (Sociocultural Research Consultants, LLC, Los Angeles, California, USA; https://www.dedoose.com). Double coding by the lead Kenyan investigator occurred at predetermined intervals (every fourth transcript; *n* = 17), with discrepancies discussed and resolved by consensus to validate the codebook and maximise coding reliability. The data was analysed using framework analysis^23^ with interpretive descriptions, both widely used analytical tools that complement each other. Framework analysis involves indexing all verbatim text, charting information from each transcript on to a series of thematic matrices and comparing between intervention and control households. The choices of thematic headings were guided by both the core concepts emerging out of the data using open coding^24^ and theoretical concepts from the design process,^25^ which were predominantly aligned with the Family Stress Model. The Family Stress Model shows how poverty and economic pressure affect the quality of interparental relationships, which affects child outcomes. Empirical support for the Family Stress Model has been established across various contexts, including diverse cultural backgrounds, family structures (i.e. two-parent and single-parent families), urbanicity (i.e. urban and rural samples) and populations in several different countries.^26,27^ Specific topics were designated as core categories; axial coding and a constant comparison approach explored the relationships between the discussion of sensitive data and contextual situations across arms. Throughout data collection and analysis, we practised reflexivity. We acknowledge that the female interviewers may have influenced gender-sensitive responses, with female participants potentially feeling more comfortable and male participants more reserved. Their trilingual proficiency reduced language barriers and enriched data collection. Their background in sociology and public health enhanced probing depth but might have led to a focus on certain aspects based on their academic training. During coding and analysis we continually examining our biases, preferences and theoretical perspectives and how those factors played a role in our understanding and interpretation of the data. All names accompanying quotations below are pseudonyms.

### Ethics approval and consent to participate

The authors assert that all procedures contributing to this work comply with the ethical standards of the relevant national and institutional committees on human experimentation and with the Helsinki Declaration of 1975, as revised in 2008. All procedures involving human participants/patients were approved by the Kenya Medical Research Institute Science and Ethics Review Unit (KEMRI/SERU/CMR/P00086/3696), and the Committee for Human Research of UCSF. Participation was entirely voluntary and had no impact on either the receipt of intervention or standard of care services at the link health facilities. Informed written consent was obtained from all participants. Adolescents who were <18 years old and not emancipated provided written assent before they participated in the study, and their caregivers provided written consent.

## Results

Three themes emerged highlighting how *Shamba Maisha* households (intervention) compared to control reduced levels of poverty and food insecurity, which may have led to the following improvements in parenting: (a) decreased parental stress; (b) reduced parental absenteeism and withdrawal; and (c) reduced harsh parenting and disciplining practices and improved caregiver–adolescent communication. Additional themes suggest that the improvement in overall household dynamics may have resulted in (a) improved psychological well-being of adolescents and (b) reduced behavioural problems, such as risk-taking, in the intervention compared to the control households.

### Income and food security

Participants reported that the intervention improved their income, and as a result, they could meet their obligations, including parenting:

‘Right now, I have money to provide for their needs because of farming. In the past, there are times that I never had even Ksh 50 and never got any help from anyone.’ (Angweng, female caregiver, intervention arm)

Similarly, participants report joining table banking groups to help save to accomplish large projects. Some also preferred paying in small instalments to acquire assets. Specifically, one adolescent narrated how the parent used to pay in small instalments to finish off buying land using farming proceeds:

‘After a short while, she joined *Shamba Maisha* where she was given farm inputs and over a period of time she managed to buy her own land. She used to pay in instalments.’ (Abungu, adolescent, intervention arm)

The *Shamba Maisha* intervention was also associated with improved food security. Participants described how the intervention resulted in their households being food secure even without having money:

‘*Shamba Maisha* has helped us a lot regarding food security in our household. Even when we do not have money, we are sure to have food. Even during drought season, we know we will eat because of the water pump.’ (Lupita, adolescent, intervention arm)

Unlike in the intervention, those in the control households expressed struggles and challenges in getting food for their family. Reports of missed and insufficient meals were common, which were attributed to lack of a steady income to purchase food. In some households, the source of income for food was money adolescent girls solicited from their boyfriends:

‘I would then call my boyfriend and tell him that we do not have food, and he would send me money. If he sends Kshs. 1000, I would go to the shop, buy flour and other foodstuffs for the family to eat during lunch time.’ (Atieno, adolescent, control arm)

One of the control households reported that the lack of food situation occasionally deteriorated to the point where they often relied on neighbours for food assistance to get by:

‘Sometimes it gets difficult to the point of borrowing food from neighbours with plans of returning; this happens once in every month or after every two months.’ (Aluoch, adolescent, control arm)

### Parental stress

Parents described how the intervention boosted their confidence and ability to meet the needs of the adolescents. This confidence facilitated conversations between parents and their adolescents on various topics, including on matters related to sexual and reproductive health. They were also confident that since they could provide for the personal needs of their girls, such as sanitary towels, men were unlikely to succeed in swaying them into sexual activities. In this regard, parents lowered the chances of their adolescent girls getting involved with men to receive money:

‘Right now I am confident that anything they need I am able to provide and that makes me more comfortable when handling those topics (sexual and reproductive health). Even if they want money, I can give them and so they don't look forward to getting money from men. They have money to buy even pads when they need them.’ (Angweng, female caregiver, intervention arm)

Compared to intervention participants, control participants seemed to exhibit a perceived disempowered position, possibly because of lack of financial resources that eroded feelings of social confidence and weakened the sense of personal efficacy that is needed to assume a firm parental role. The unfavourable ‘material’ environment acted as a constant reminder of parents’ ‘failures’ that resulted in feelings of hopelessness.

Caregivers in the control arm had feelings of failing and shame when they could not provide for their children and had to keep borrowing:

‘I have difficulty in that if I don't get these things; when I arrive at home without them my child normally feels pained in the heart because she can see that her peers have them and so it becomes difficult for me … I also feel hurt because I feel like I have wronged her by not being able to meet her needs.’ (Ajuoga, female caregiver, control arm)

‘I used to look for sanitary towels at home in vain. Therefore, I had no idea how she managed to survive during her periods. I also felt bad being helpless as she is only a girl; she'd also feel pain seeing me down, looking at the empty house, no money.’ (Aoko, female caregiver, control arm)

### Parental involvement (withdrawal/absenteeism)

For intervention households, the financial security empowered caregivers to make their own decisions about where and how to spend time, in or away from home, and enabled caregivers to provide more consistent parenting. Parents in the intervention households commented not only on having more time with their children but also on being emotionally present when they were with their children as opposed to constantly being away working or worrying about the next meal:

‘Before we joined *Shamba Maisha*, we were never close and I had no time to sit down for a catch up with any of them because I had so much stress thinking of how the household is going to get food daily. I never explained to them why I was so distant either so they also avoided me. I would leave very early and when I return, I wouldn't find them, and whenever I scolded them or her specifically about loitering around, she would ask me where I am from myself? And they were right because I was never there. After I joined the programme … I sat them down and explained to them where I was from and what had been happening.’ (Abote, female caregiver, intervention arm)

In control households, the daily challenges of providing for the family were apparent, leading to a compromise in parental involvement in the children's affairs. In certain cases, caregivers even avoided speaking to their adolescents, particularly after a misunderstanding:

‘When I wronged her, she used to avoid talking to me. When she is angry, she goes straight to her bedroom and keep quiet. She would not talk to me until I initiate a conversation. I would attempt asking her a question just to gauge if she will talk to.’ (Achieng, female caregiver, control arm)

Working together in the farms helped parents have better control of their children. It provided an opportunity to teach them a skill for future income generation and independence as well as kept them occupied and less likely to engage in high-risk sexual behaviour as demonstrated by this father–daughter dyad:

‘It has helped me by: one; involving them and using their time positively. By this I mean, instead of them just roaming around because I stay near the lake, during weekend I take them with me to the shamba [farm], they work there and help me generate the income. This helps me keep them out of danger [sexual risk]. At the same time, I will have instilled that culture of income-generating activity so that even if she is not at home, she knows that there is a way of generating income.’ (Ang'ina, male caregiver, intervention arm)

### Parenting styles

Emotional distress also manifested as harmful caregiving practices that included lack of warmth and support and displays of aggression or hostility by parents. Parents from the intervention reported less hostility and enhanced warmth and support compared to those from control households. This in turn enhanced the caregiver–adolescent relationship, which is critical when it comes to laying a platform for discussions about sexual and reproductive health and hence promote responsible behaviour among adolescents:

‘My mother used to be very angry, she would even hurt you when she is caning you … She would say, “You can't see the way I am suffering and you just want to add unto my sorrows” … after joining *Shamba Maisha*, she would just threaten that she will lock me outside but she never did it … She is in more control of her emotions. She still quarrels when something is wrong but she is not as harsh as she used to be before she joined *Shamba Maisha* … The change has been caused by the fact that she is ok financially … I think she was harsh because she did not have a stable job and thus she didn't get enough income.’ (Abungu, adolescent, intervention arm)

R: ‘I am not as harsh as I used to be before; I am less stressed and not frustrated or blame them (the adolescents) for anything. I think my way of disciplining these days is rather rational and in an appropriate way.’

I: ‘Why were you so harsh?’

R: ‘I had too much burden on me and I felt like I was losing it sometimes, hence I had no time to talk to the children, I just beat them up. Nowadays I talk to them and explain why I am actually disciplining them and encourage them to stop such behaviours.’ (Abote, female caregiver, intervention arm)

In contrast, caregivers in the control group encountered ongoing difficulties. They expressed that their methods of disciplining their children were unsuccessful, failing to produce the desired outcomes:

‘They (methods of discipling) weren't effective because she never calmed down to apologise to me when she does something wrong. Sometimes I am the one who is forced to calm down and have mercy on her and leave the house key behind.’ (Akiki, female caregiver, control arm)

### Parent–child communication

With the economic empowerment from the intervention, parents had more time with their adolescents. They used this time to check in with their children, including on issues related to sexual reproductive health. Narration from the mother–daughter dyad below sums up this experience:

‘I can nowadays get time to sit with her down and start talking about these topics (sexual reproductive health); in the past, before I joined *Shamba Maisha*, I would come back home late and tired thus no opportunity to talk. However, after being part of the intervention I find it easy to provide for my family and I am able to have extra time to chat with my children.’ (Aunda, female caregiver, intervention arm)

‘Long ago she never used to listen when I was talking to her because she would be absent minded, currently we can hold a conversation with her and she will listen and advise me. Moreover, if I ask her for something, she gives me. Before she joined *Shamba Maisha*, I never used to experience such with her. She always lacked. In addition, before *Shamba Maisha*, she rarely got time to sit down with me and discussing anything.’ (Abungu, adolescent, intervention arm)

For some parents in the control households, it was challenging to find time to talk with their adolescent girls either because they are away most of the time or the girls are busy with school work:

‘I hardly get time to talk to her nowadays because I am very busy; she is always at school throughout and on Saturdays she probably does laundry and other house chores and on Sunday, we leave church at 10 a.m. and then she gets back to her chores as she prepares for school on Monday.’ (Anindo, female caregiver, control arm)

However, a segment of control households noted a softening in the way their adolescents communicated between the time of enrolment and the time of the interview:

‘She used to be very rude. She could answer you not seeing that she is addressing an elder person … I can now see that she toned down. Ever since spending the two nights away she has been calm.’ (Amollo, female caregiver, control arm)

### Adolescent psychological well-being and behaviour outcomes

As a result of households achieving more financial security, adolescents in the intervention expressed feelings of safety and improved psychological well-being and reduced behaviour problems, such as aggression or other high-risk behaviour. For instance, adolescents from intervention households exhibited improved psychological well-being associated with better parenting practices resulting from economic empowerment and food security. With school fees and other needs met, adolescents reported peace of mind. Similarly, parents’ stable mental health owing to increased income and availability of food gave adolescents confidence to approach them for counsel without worrying about their moods:

‘I'm free these days and don't have the fear I initially had. Nowadays I can sit with mother and even tell stories. [In the past] she was quick to fight and inflict pain on me at the slightest provocation and it isn't easy to be free or close to such a person … Back then I would even get shy to ask her for the sanitary towels; we weren't very close.’ (Atiende, adolescent, intervention arm)

‘Before, I never used to be very jovial but now I can have time with my mother and share things and laugh together… [Before the intervention] She was never there, she used to leave very early in the morning and come back late in the evening and very tired.’ (Aliech, adolescent, intervention arm)

Parents reported that adolescents’ behaviour changed after the intervention. They attributed this change to parents being able to meet the adolescents’ needs owing to improved household income. This behaviour change is reported by both caregivers and adolescents:

‘She used to leave home most of the times in the morning and return at about 9 p.m. before I joined *Shamba Maisha*, but nowadays, she barely leaves home. Whenever I would ask about her whereabouts, she would always get angry but since I joined *Shamba Maisha*, she has really changed; she always asks for permission before leaving home.’ (Atich, male caregiver, intervention arm)

‘ … It [current discipline method] is good because before when I was that rude girl, she would inflict pain on me by caning to make me stop a particular behaviour but it never worked; I would repeat the same mistake on and on. Now she just talks to me … Her verbal means of discipline now is very effective.’ (Aluoch, adolescent, intervention arm)

Adolescents reported that they had a strong desire to have sexual relationships to gain access to materials and financial resources they did not have because of financial challenges caregivers were struggling with. However, the desire reduced when finances improved after joining the *Shamba Maisha* intervention:

‘I had a great desire to have a boyfriend when my mother was struggling financially because I didn't want my friends to disrespect me. During that time, my friends had boyfriends who were supporting them financially, some had rented for them houses and some were even married. So I wanted to have a boyfriend to uplift our household finances … The desire was still there but not as much because our family finances improved after my mother joined *Shamba Maisha*.’ (Abungu, adolescent, intervention arm)

‘Many parents cannot afford to meet the needs of their children and so they fall prey to men who promise them financial support to meet their needs. However, their parents should talk to them so that they understand the situation at home and let them know when they will be able to meet their needs.’ (Angweng, female caregiver, intervention arm)

## Discussion

We set out to assess the pathways for how an agricultural and livelihood intervention may have affected parenting practices and caregiver–adolescent relationships. We found that our agricultural intervention strengthened the economic security of households, leading to positive parenting practices and enhanced caregiver–adolescent relationships, depicted in decreased parental stress, reduced parental absenteeism and withdrawal, reduced harsh parenting and disciplining practices and improved caregiver–adolescent communication and relationship. We also found that positive caregiving practices together with enhanced caregiver–adolescent relationships in turn resulted in improved adolescent psychological well-being and behaviour.

Our findings are consistent with other studies that have shown that strengthening economic interventions that assist low-income families to accumulate economic resources improve the economic well-being of families. These studies also show that reducing parenting stress that originates from caregiving roles can reduce child–caregiver dysfunctional relationships and improve the overall family functioning. ^28^ Food insecurity and poverty can affect the quality of parenting and parental stress to negatively affect parenting practices, thereby adversely affecting adolescent behaviour and mental health. ^29^ In a study in Burkina Faso, female caregivers described how an economic strengthening intervention helped them reclaim their role as mothers.^30^ They explained that scarcity of resources and the inability to meet the demands of their children strained the relationships, leaving parents with guilt and children resentful. In our study, the improved food security and reduced poverty within the households reduced parental stress and improved parental confidence, resulting in improved quality of parenting and a reduction in the occurrence of negative caregiving practices. Our study builds on findings from economic empowerment interventions by explicitly addressing how improvements in food insecurity, arguably one of the most important forms of poverty-related stress, improved family relationships and adolescent health.

Our findings that harsh and inconsistent parenting led to displays of behavioural problems such as aggression by adolescents have been supported by other research. For example, studies show that that harsh, cold and inconsistent parenting increases the risk that adolescents will develop both externalising disorders (behavioural problems such as aggression) and internalising disorders (anxiety and depression),^30,31^ in turn increasing risk-taking behaviours.^31^ Adolescents highlighted that harsh punishment, inconsistent discipline and poor monitoring and supervision interfered with their mental health, echoing the findings of previous research.^31^ In addition, corporal punishment, an aspect of harsh discipline, increases the risk for behavioural problems.^32^ On the other hand, positive parenting – when parents are warm and affectionate and have positive interactions with their children – strengthens the caregiver–adolescent relationship, allows opportunities for better parental monitoring and promotes good outcomes for children.^33^ Positive parenting is associated with high self-esteem, future optimism and school satisfaction,^34^ which are all important for an adolescent's transition to adulthood. Good mental health and psychological well-being supports resilience and healthy behaviours that shape long-term positive health outcomes. In our study, parents noted that when their economic status elevated, their mental health was improved, and this contributed to them being less harsh and warmer towards their adolescent girls. In turn, the adolescents also became happier, communicated better and exhibited more self-disciplined behaviour. Further, studies have shown that improvement in incomes similar to that reported in our study was associated with reductions in caregivers’ emotional hostility and instability, psychotic symptoms and interpersonal sensitivity, thus promoting positive parenting.^35^

Heads of poor and food insecure households are known to spend long hours working away from their families, which weakens family connections and relationships as a result. The lack of time spent with adolescents and not being able to meet their needs can disrupt parental–adolescent connectedness, which is an essential factor in parents’ ability to control or regulate the behaviour of their adolescents.^23,24^ Parents in our study were unable to monitor or control the behaviour of their adolescents when they spent many hours away trying to earn income, which in turn affected their adolescents’ sexual relationships and practices. This was most notable in cases where caregivers where neither available to counsel the adolescents nor provided for their needs. This finding is corroborated by several other studies in sub-Saharan Africa.^36^ The *Shamba Maisha* intervention made parents more available and accessible at home because it improved financial and food security. Parents therefore had the opportunity to spend more time with their children. This gave them a better chance to monitor and guide their adolescents. The time parents and adolescents spent together on the farm working was also a way of positively engaging adolescents. There is evidence that this kind of caregiver–adolescent interaction and bonding strengthens caregiver–adolescent relationships and gives parents the opportunity for better monitoring and guidance of the adolescent.^37^

Our study had several limitations. First, social desirability bias may have contributed to underreporting of conflict between parents as an aspect of negative parenting. While other studies have associated parental conflict with food insecurity and poverty, there is a possibility that our participants could have underreported these themes because of the shame that comes with sharing that information. Second, in our conceptual model, we present a simplified relationship among food insecurity, economic stress and parenting for the purposes of our data analysis, and reporting even the true relationship may be complex and multi-stranded. For further qualitative analysis of the impact of this programme, consider the recent publication by Onono et al,^38^ which explores the links between household-level income-generating agricultural interventions and the psychological well-being of adolescent girls in HIV-affected households in south-western Kenya. This study offers additional qualitative insights, particularly on themes such as the psychological impact of food insecurity on adolescents, which complement the findings in this paper. Third, given that our study was done at the end of the intervention implementation, our study could have been subject to recall bias. To counter this bias, future qualitative studies could benefit from longitudinal designs, which do not require participants to recall events that happened over longer time frames. Although the qualitative data collectors were not part of the intervention implementation team and were unknown to participants until the time of the interview, there was still a risk of participants over-reporting or exaggerating benefits associated with the intervention. Lastly, given the research was conducted exclusively in the south-western region and focused on qualitative analysis within this specific locale, it is likely that the findings may not be applicable to a broader population. Instead, they can only be appropriately compared to outcomes from comparable contexts and settings.

In conclusion, we have shown that food insecurity and family poverty negatively affect the quality of parenting practices, caregiver–adolescent relationships and eventually adolescents’ mental health and high-risk sexual behaviour. These findings provide important considerations to policy makers and programmers to target the root causes of food insecurity to improve parenting practices and experience and mental health and prevent high-risk sexual behaviour among adolescents. Results from this study extend the emerging body of work on food insecurity and caregiver–adolescent relationships by demonstrating how an income-generating agricultural intervention can mitigate the non-nutritional consequences of food insecurity on parenting practices and adolescent behaviour. Interventions to improve young people's sexual and reproductive health should recognise the structural effects of parenting and we recommend adding structural interventions focused on reducing poverty and food insecurity. An ecological strategy for such interventions would attempt to affect the changing socioeconomic landscape of parenting at different levels. Intentional parenting programmes could innovate and assist parents to identify positive parenting practices that foster healthy adolescent development. HIV programmes should consider adding parenting interventions and youth group meetings to improve downstream outcomes, such as improved mental well-being and elimination of high-risk sexual behaviour and unplanned pregnancy.

## Supporting information

Onono et al. supplementary material 1 Onono et al. supplementary material

Onono et al. supplementary material 2 Onono et al. supplementary material

Onono et al. supplementary material 3 Onono et al. supplementary material

## Data Availability

Data cannot be shared publicly because this study was conducted with approval from the Kenya Medical Research Institute (KEMRI) Scientific and Ethics Review Unit (SERU), which requires that we release data from Kenyan studies (including de-identified data) only after they have provided their written approval for additional analyses. As such, data for this study will be available upon request, with written approval for the proposed analysis from the KEMRI SERU. The application forms and guidelines can be accessed at https://www.kemri.org/seru. To request these data, please get in touch with the corresponding author or the KEMRI SERU at seru@kemri.org.
