# Effect of improving food security on parenting practices and caregiver–adolescent relationships: qualitative findings of an income-generating agricultural intervention in rural Kenya – CORRIGENDUM

**DOI:** 10.1192/bjo.2025.38

**Published:** 2025-04-22

**Authors:** Maricianah A. Onono, Lila Sheira, Edward A. Frongilio, Gladys Odhiambo, Pauline Wekesa, Amy Conroy, Elizabeth A. Bukusi, Craig R. Cohen, Sheri D. Weiser

In the original publication of this article, reference 19 was incorrectly listed as:

“Cohen CR, Grossman D, Onono M, Newmann S, Burger RL, Dith C, et al. Integration of family planning services into HIV care clinics: results one year after a cluster randomized controlled trial in Kenya. PLoS One 2017; 12(3): e0172992.”

Instead, this should have been:

“Weiser SD, Bukusi EA, Steinfeld RL, Frongillo EA, Weke E, Dworkin SL, Pusateri K, Shiboski S, Scow K, Butler LM, Cohen CR. Shamba Maisha: randomized controlled trial of an agricultural and finance intervention to improve HIV health outcomes. AIDS. 2015 Sep 10;29(14):1889-94. doi: 10.1097/QAD.0000000000000781. PMID: 26214684; PMCID: PMC4573846.”

This has now been updated in the article and this corrigendum published.
