# Supplementary material for: Effect of improving food security on parenting practices and caregiver–adolescent relationships: qualitative findings of an income-generating agricultural intervention in rural Kenya
Source: BJPsych Open. 2024 Dec 26;11(1):e10. doi: 10.1192/bjo.2024.802 (PMC11733493; doi:10.1192/bjo.2024.802)
Supplement: Onono et al. supplementary material 1 — Onono et al. supplementary material [file S2056472424008020sup001.doc]

**Qualitative Study Guide: Understanding the potential impact of a household level income generating agricultural intervention on adolescent sexual behavior and psychological well-being and the use of sexual and reproductive health and HIV services**

**Please collect PAGE 1 for each interviewee**

| **RESEARCHER NAME:__________________ ID:**  **DATE:**  **Month Date Year**  **DISTRICT NUMBER: INTERVIEW NUMBER:** | . |
| --- | --- |

**INTRODUCTION [AFTER GETTING CONSENT FROM PARTICIPANT]**

Thank you for meeting with me. We are talking to adolescent girls who have been living in a household that has been a part of *Shamba Maisha* program. Today I would like to hear more about the potential impact of a household level income generating agricultural intervention on your behavior, psychological well-being and your uptake of sexual reproductive health and HIV services. We will begin by talking in general about adolescent girls and sexuality. We will then go deeper into your experience with the *Shamba Maisha* project as a study participant and talk about food security, wealth, health, parental communication and your household functioning. Lastly, we will ask about where you receive or can receive sexual reproductive health services. I am also interested in any recommendations/suggestions you have for how we can improve the Shamba Maisha project so that it benefits adolescent girls like you

Don’t worry if you can’t remember some details of the program - I am more interested in your overall experiences. There are no right or wrong answers to these questions and I am most interested in your detailed explanations and stories. In fact, I want you to speak most of the time and me to speak very little. This is your chance to tell me how you feel. Also, you are free to decline to answer these questions. Please remember that anything you tell us is confidential and will not affect your involvement in receiving health care or at any health facility with the program in any way.

We will collect the information, keeping participants anonymous. When responding to questions, please do not identify yourself or anyone else by name.

Do you have any other questions about this project or the consent form?

[If not], OK…let’s go ahead and get started. Please stop me if you want to take a break, or if would like any part of interview to be unrecorded.

**Section A:**  **ICEBREAKERS: [ASK SECTION ALSO TO INTERVENTION AND CONTROL PARTICIPANTS]**

**[Note to interviewer: the purpose of this section is to begin engaging the participant in conversation and gain rapport.]**

I would like to begin by asking several questions about you:

1. How are you doing today?
2. How old are you?
3. Have you ever attended school?
4. What is the highest standard/form in school that you have achieved?

**SECTION B: FOOD SECURITY**

**[Note to interviewer: the purpose of this section is to explore perceived changes in food adequacy, dietary diversity and frequency of meals.]**

1. To get started, I would like to hear first about your food situation. Tell me more about your food situation prior to joining Shamba Maisha? **[FOR CONTROL PARTIPANTS, ASK THIS QUESTION ABOUT CURRENT FOOD SITUATION]**
   1. Probe on challenges with access to food, and food shortages
   2. Probe on challenges with diet quality and diversity of available foods
   3. Probe on relying on charity or others for food
2. Since your household joined the Shamba Maisha study, could you talk about changes, if any you have experienced regarding your own food situation? [PROBE FOR NARRATIVE STORIES]
   1. Probe on changes in number of meals eaten in a day
   2. Probe on changes in frequency of food shortages, particularly during dry season
   3. Probe on changes in diet/variety of foods served and whether changes limited to dry season.
   4. Probe on changes in distribution of food among household members (especially to the specific adolescent and other adolescents in the house?)
   5. Probe on changes in distribution of food based on gender (male and female adolescents).
   6. Probe on reasons for change in food situation.
   7. Probe on other continued food-related challenges despite being in the program.

FOR INTERVENTION PARTICIPANTS:

1. How do you think the Shamba Maisha intervention affected availability of food for you as an adolescent girl?
   1. Probe on changes in distribution of food based on gender (male and female adolescents.
2. How do you think the Shamba Maisha intervention could be improved to further improve access to food for adolescent girls like you at the household level?

**SECTION C: HOUSEHOLD WEALTH:**

**[Note to interviewer: This section seeks to explore adolescent’s perceptions on access to socially perceived household necessities e.g. toiletries, sanitary towels, clothes, and shoes, as well as school uniforms, stationery, and fees**.**]**

1. Now I would like to hear about household items in your house such as toiletries, sanitary towels, clothes, shoes, uniforms, school stationery, school fees. Tell me more about the availability of these necessities prior to joining Shamba Maisha? **[FOR CONTROL PARTIPANTS, ASK THIS QUESTION ABOUT CURRENT SITUATION]**
   1. Probe on challenges with access to toiletries including sanitary towels, clothes, and shoes. [IF IN SCHOOL:] Probe on challenges in purchasing school uniforms, school supplies, and fees
   2. Probe on challenges with quality and variety of available toiletries including sanitary towels, clothes, and shoes, as well as school uniforms, stationery, and fees
   3. Probe on relying on charity or others for purchasing toiletries including sanitary towels, clothes, and shoes for household
   4. Probe on relying on charity/help from others to pay fees, staying in school, purchase school uniforms and stationery
   5. Probe on relying on charity/help from others to access items such as soaps, lotions, make up, perfumes
2. Since your household joined *Shamba Maisha*, could you talk about any changes you have experienced in terms of access and purchase of toiletries, sanitary towels, clothes, shoes, uniforms, school stationery, and school fees? [PROBE FOR NARRATIVE STORIES]
   1. Probe on changes in income to ensure basic needs for her
   2. [IF IN SCHOOL] Probe on changes in income to ensure that she was kept in school and not missing school due to lack of fees, uniform, stationery, sanitary towels
   3. Probe on changes in proportion of income within the family allocated to her and other adolescents in the house
   4. Probe on changes in having to give up basic needs due to a tight financial situation
   5. Probe on changes in relying on charity/help from others compared to before intervention
   6. Probe on other possible explanations for the changes
3. Since your household joined *Shamba Maisha,* could you talk about any changes you have noticed in your general life at home or with friends?
   1. Probe on changes in need for her to work to supplement household income before joining the program
   2. Probe on changes in family involvement (likelihood to spend more time in the household vs. visiting others, more social, spoken
   3. Probe on changes in increasing social networks e.g. had more friends, more visitors, more outgoing
4. Since your household joined Shamba Maisha, can you speak about any changes you have noticed in your schooling and education? [IF IN SCHOOL]
   1. Probe on availability of school fees
   2. Probe on changes in going, staying or skipping school (due to availability of fees or having not to work)
   3. Probe on changes in educational performance (ASK IF *Shamba Maisha* had an effect on education for intervention participants)
   4. FOR INTERVENTION PARTICIPANTS: Probe on mechanisms for change (ex: more income for school fees, better concentration due to less stress or due to less food insecurity etc…)
5. Since your household joined Shamba Maisha, can you speak of any changes you have noticed in your mental or physical health
   1. Probe on changes in physical health
   2. Probe on changes in mood (happiness, symptoms of depression)
   3. **FOR INTERVENTION PARTICIPANTS**: Probe on mechanisms for change (ex: less stress, improved income, improved diet quality, improved family relationships etc…),

**[CONTROL PARTICIPANTS SKIP TO SECTION D: ENABLING CARING ENVIRONMENT]**

1. **INTERVENTION PARTICIPANTS ONLY:** Based on what you just said about the impact of *Shamba Maisha* in your life*,* how do you think the *Shamba Maisha* intervention could be improved to further improve your belief in your own ability to succeed and plan for your future?
   1. Probe on ways SM can further improve schooling.
   2. Probe on ways that SM can further improve education.
   3. Probe on ways that SM can further improve your physical health.
   4. Probe on ways that SM can further improve your mood and mental health.

**SECTION D: ENABLING CAREGIVING ENVIRONMENT**

**[Note to interviewer: This section seeks to explore concepts around involvement (i.e., spending time with and showing interest in the adolescent), quality of communication (i.e., parent empathy and conversation across situations). We are also interested in changes in parenting monitoring: 1) positive parenting, 2) consistent discipline, and 3) good supervision]**

1. What are typical evenings/weekends like for your family?
   1. Probe on leisure activities that they involve the adolescent girl.
   2. Probe on activities that the adolescent girl is happy/unhappy/ interested/disinterested in.
2. When you are having issues with school or are having a bad day is there any family member who is primarily concerned? Tell me more about this.
3. How do you have discussions with your caregiver when you have any psychosocial issues like feeling low or afraid?
4. How does your caregiver or parent discipline/supervise you?
   1. Probe on how the caregiver checks on the way the adolescent girl(s) behaves or feels
   2. Probe on the caregiver’s style of discipline/supervision (how they reward good behavior and manage bad behavior, caution on any risky behavior)
   3. Probe on whether the adolescent thinks it is effective
   4. Probe on how the adolescent girl feels about the way they discipline?
   5. Probe on how the adolescent feels their caregiver’s style works, is there anything they would change?
5. Since your household joined *Shamba Maisha*, could you talk about any changes you have experienced in how you relate with your caregiver/parent?
   1. Probe on changes in the way the caregiver monitors/supervises the adolescent
   2. Probe on changes in the way the caregiver disciplines the adolescent (e.g. stricter vs accommodating, physical vs verbal, withdrawing or giving affection or gifts.)
   3. Probe on changes in how the caregiver rewards good behavior and manages bad behavior, cautions on any risky behavior)
   4. Probe on changes in the communication between them and caregiver? (include quality and frequency, more or less involvement of third parties e.g. aunts or older sisters)
6. Since your household joined *Shamba Maisha,* have you had conversations with your caregiver about sexual and reproductive health issues? Why or why not?
   1. Probe: was this happening before Shamba?
   2. [**For those who discuss sexual and reproductive health** **issues with their caregivers:**] What topics do you discuss with them?
   3. Probe to find out how easy/hard it is to discuss these topics; which topics they consider a priority
   4. Probe specific messages delivered on this topic.
7. Could you talk about how you perceive your caregivers/parent comfort level in talking about topics of sexual and reproductive health?
   1. Probe on whether and how this has changed in the past year.
   2. Probe on frequency of conversations and variety of topics.
8. Could you talk about your confidence in being able to have a conversation on bodily changes i.e. puberty right now?
   1. Probe on the confidence now compared to that from before you enrolled in the program.
9. **INTERVENTION PARTICIPANTS ONLY [FOR CONTROL PARTICIPANTS, SKIP TO SECTION E]:** How, if at all, do you think the *Shamba Maisha* intervention has affected your relationship with your caregiver?
   1. Probe on mechanisms for change (ex: reduced stress from less FI, more household wealth and assets, increased time spent together e.g. working on the farm together or parents doing less side-hustles)
10. How, if at all, do you think the *Shamba Maisha* intervention has affected your communication about sexual health and bodily changes with your caregiver? (Probe on mechanisms such as reduced stress from less FI, more household wealth and assets, increased time spent together e.g. working on the farm together or parents doing less side-hustles)
11. What other kind of intervention would you recommend to be paired with Shamba Maisha that would be improve your relationship and communication with your caregiver
    1. Probe – direct counseling of parenting and adolescents at home, Parenting classes on how to handle adolescents, communication classes

**SECTION E: SEXUAL HISTORY**

**[Icebreakers about community norms:]**

1. Around when do girls start becoming sexually active your community? By sexually active I mean engaging in any type of sexual activity (kissing, touching, or more) with a man.
2. How do girls usually meet these men? Where do they continue to meet while they are sexually involved?

**[INTERVENTION:] Thank you: I want us now to talk about your sexual experiences since your household became involved in *Shamba Maisha*.**

**[CONTROLS:] Thank you: I want us now to talk about your sexual experiences over the past two years.**

1. Have you had at least one partner with whom you were sexually involved? [**IF SEXUALLY INEXPERIENCED, I.E. ANSWERED NO TO QUESTION 3, SKIP TO QUESTION 15]**
   1. Probe: Have you ever been sexually involved with a man?
   2. Probe: What influences your partner choice/selection? (able to provide food (snacks), money, airtime or just access –availability- men are available during holidays)
2. Where do you meet your partners? How long have you generally known / seen your partner before sex?
3. To what extent do negotiations about sex take place?
   1. Probe: Who leads the discussions? What is said?
   2. What normally leads to sex?
   3. Reasons for engaging in sex
4. Do you pay or get paid for sex? Receive/give gifts?
5. Have you had partners who you haven't had sexual intercourse with? What influences whether or not they become sexual partners?
6. Do you have multiple partners? Times when you are seeing more than one person?
7. How would you describe your relationships? Do they tend to be casual? Are they serious?
8. Are you fearful of others finding out about your activities? Who? Why?
9. **[ASK *SHAMBA* INTERVENTION PARTICIPANTS; CONTROLS SKIP TO QUESTION 16]:** How, if at all, do you think the *Shamba Maisha* intervention affected your number and choice of partners and sexual activities when compared to before the project?
   1. Probe about role of adequate food
   2. Probe about role of increased household wealth
   3. Probe about role of improved caregiving environment
10. How, if at all, do you think the *Shamba Maisha* intervention affected your confidence in negotiating for sex?
    1. Probe for increase or decrease or no effect
    2. Probe for mechanisms of change (ex: role of reduced food insecurity, increased household wealth and the improved caregiving environment etc…)
11. How do you think the *Shamba Maisha* intervention could be improved to further improve your confidence in negotiating for sex?
12. What do you think can be added to the Shamba Maisha intervention to help you make healthy choices regarding sex life and health? (Probe on interventions such as 1) cash or non-cash conditional/unconditional transfers, life skills training, training in negotiation, school vs home vs community based interventions, buddy (mentor-sister) systems), mobile technology like text reminders or chat rooms)

**[WHEN COMPLETE, SKIP TO QUESTION 16]**

**[QUESTIONS FOR SEXUALLY INEXPERIENCED GIRLS IN INTERVENTION AND CONTROL]**

1. When do you think it is ok to start having sex?
   1. Probe: When is it too early to start having sex?
   2. Can you think of a reason why girls in your community have sex before they are really ready?
   3. Do you think girls in your community are pressured to have sex before they are ready? How or how not?
   4. Why do you think you haven't had sex yet?
   5. Do you feel ready? Why? Why not?
   6. When will the time be right for you?
   7. Do you feel under pressure to have sex? From whom? How does this make you feel?
   8. Do you feel pressure not to have sex? From whom? How does this make you feel?
      1. How have you resisted the pressure(s) to have sex?
      2. Probe for the role of the families wealth status/security in enabling her avoid/delay sex
      3. Probe for the role of the accessibility to food in enabling her avoid/delay sex
      4. Probe for the role of parental monitoring and supervision in enabling her avoid/delay sex
   9. What do you imagine the first time to be like?
      1. Probe: With whom? Age? What stage in the relationship?
      2. Type of partner?
      3. Do you plan to use contraception?

**SEXUAL RISK TAKING [ASK FOR THOSE WHO ARE SEXUALLY ACTIVE—IF NOT SKIP TO SUB-SECTION 2: CONDOMS; QUESTION 26]**

**[Sub-section E1: Contraception]**

**Thinking now about the most recent sexual encounter during the time since your household became involved in Shamba Maisha:**

1. Can you share with me what you know about contraception among young people?
2. Have you used contraceptives to prevent pregnancy? If so, which one(s)?
   1. Probe: Where were the methods of preventing pregnancy obtained from?
   2. Who provided them? How often did you use them?
   3. Which method did you use most commonly? Why did you use the method you chose?
   4. Was your partner involved in the decision making process? If so, how?
3. Did either of you have any problems / barriers to obtaining protection/contraception?
   1. Probe: What were they? Were they overcome? How? Why not?
4. Who generally provides these contraceptive methods? Whose responsibility is protection?
5. Were there times when no contraceptive method was used? Please tell me more about the circumstances around one such time.

**FOR INTERVENTION PARTICIPANTS ONLY—CONTROLS SKIP TO QUESTION 26]**

1. How do you think, if at all, the *Shamba Maisha* intervention affected your feelings about, and knowledge of various types of contraception?
2. How do you think the *Shamba Maisha* intervention affected your confidence in negotiating for use of contraceptives?
3. How do you think the *Shamba Maisha* intervention could be improved to enhance your feelings about, and knowledge of, various types of contraception.
4. What other kind of intervention would you recommend to be paired with Shamba Maisha that would improve your ability to negotiate and use contraceptives correctly and consistently. (Probe on interventions such as 1) cash or non-cash conditional/unconditional transfers, life skills training, training in negotiation, school vs home vs community based interventions, buddy (mentor-sister) systems), mobile technology like text reminders or chat rooms or contraceptives on demand services)

**[Sub-section E2: Condoms]**

1. Can you share with me some of what you know about condom use in your community?
   1. Probes:
      1. How accessible is it to get condoms?
      2. Who usually decides about condom use within a couple?
      3. What do people in general think about condoms?
2. **[IF EVER SEXUALLY ACTIVE; OTHERWISE SKIP TO QUESTION 30 IF INTERVENTION AND 34 IF CONTROL]** What has been your use of condoms been like with your last partner? Why did/do you use condoms? In what instances did you not use condoms? How did you decide when and when not to use condoms? Who decided? Did you or partner influence when the condoms were /were not to be used?
3. Who generally provides these methods? Whose responsibility is protection?
4. Were there times when no protection was used?

**[FOR INTERVENTION PARTICIPANTS]**

1. How do you think, if at all, the *Shamba Maisha* intervention affected your opinions about and use of, condoms?
2. How do you think the *Shamba Maisha* intervention could be improved, if at all, to improve your confidence in negotiating for condom uses? What about to enhance your feelings about, knowledge of, and use of condoms?
3. How do you think the *Shamba Maisha* intervention could be improved, if at all, to further improve your confidence in negotiating for condom use?
4. What other kind of intervention would you recommend to be paired with Shamba Maisha that would be improve your ability to negotiate and use condoms correctly and consistently

**[Subsection E3: Risk perception [CONTROLS AND INTERVENTION]]**

1. What does it mean to be at risk of HIV, STIs, and or pregnancy?
2. In general, do you consider yourself to be at risk of HIV, STI or pregnancy?
   1. Probe: why or why not?
   2. Probe: Have you ever been pregnant? What happened? What did you do? How did you feel? How did other people react? Has it changed your behaviour?
   3. Probe: How would you feel if you found out you were pregnant? What would you do? Why?
3. Have you had a STI or symptom? Have any of your partners had a STI or symptom? What happened? What did you do? How did you feel? Has it changed your behaviour?
4. Have you ever had an HIV test? Have any of your partners had an HIV test? Why? Why not?
5. Have you ever asked a partner to have a test? Why? Why not?

**[Subsection E4: PrEP [CONTROLS AND INTERVENTION]]**

1. Can you tell me what you know about PrEP, also known as pre-exposure prophylaxis, also known as the pill you can take to avoid getting HIV if you don’t already have it?
   1. Probe: Where did you learn about it?
   2. Probe: What are the opinions in your community about it? What are your opinions about it?
   3. Probe: Do you know anyone who takes it? What do they say about it?
   4. Probe: Do you think this is something you would ever be interested in taking in the future? Why or why not?
   5. Probe: Is this something you would be interested in taking in right now? Why or why not?

**[INTERVENTION PARTICIPANTS CONTINUE; CONTROLS SKIP TO SESCTION F: SEXUAL AND REPRODUCTIVE HEALTH CARE SEEKING ]**

**[Sub-section E5: Enhancements to Shamba Maisha]**

1. How do you think the *Shamba Maisha* intervention affected your own perceived risk for pregnancy, STIs and HIV?
2. How do you think the *Shamba Maisha* intervention could be improved to enhance your perceived risk for pregnancy, STIs and HIV?
3. What other kind of intervention would you recommend to be paired with Shamba Maisha that would be improve your ability to avoid getting HIV and AIDS?

**SECTION F: SEXUAL REPRODUCTIVE HEALTH CARE SEEKING**

**[Interview note: The purpose of this section is to learn more about where adolescent girls access HIV, STI and pregnancy testing, condoms or family planning methods.]**

**Ok, now I’d like to ask about one more topic area…**

1. Can you list for me all the places young women are able to visit and people they can talk to, to find out about sex, contraception, PrEP, and/or STIs?
2. Have you ever gotten information from one of these places?
3. Do you think the sources you just mentioned are good ways for girls to learn about contraception and condom use? Why or why not?
4. Which are the best sources for this information? Why?
5. How do adolescent and young women usually find out about services?
6. What do you think are the most important features of a sexual health service for young people?
   1. Probe: What do you think are the essential elements of a service?
   2. Probe: What will make young people go?
   3. Probe: Are there differences in the needs of young men and women? How can they both be provided for?
   4. Probe: What barriers are there for accessing these services?
7. Where do you think adolescent and young women’s sexual health services should be held (location)? Why? Who should provide the information and advice?
8. Can you think of 3 words which are the most important to use when advertising and promoting sexual health services for adolescent and young women?
9. How do you think the services in your locality could be improved upon? What do adolescent and young women in your area need?

**[INTERVENTION PARTICIPANTS ONLY, CONTROLS SKIP TO CONCLUSION]**

1. How do you think the *Shamba Maisha* intervention affected your confidence to seek these sexual and reproductive health services?
2. How do you think the *Shamba Maisha* intervention could be improved to further improve your confidence to seek these sexual and reproductive health services?
3. What other kind of intervention would you recommend to be paired with Shamba Maisha that would be increase your confidence to seek these sexual and reproductive health services promptly and consistently

**SECTION G: CONCLUSION**

1. Are there any other topics that you would like to discuss?
2. Is there something you started to say about any topic that you did not get to elaborate on?
3. May I read through this interview guide quickly and make sure we have spoken about everything?

Thank you very much for your time. Do you have any questions about this interview before we end today?

**INTERVIEWER’S OBSERVATIONAL NOTES (Please write here):**
