# Supplementary material for: Effect of improving food security on parenting practices and caregiver–adolescent relationships: qualitative findings of an income-generating agricultural intervention in rural Kenya
Source: BJPsych Open. 2024 Dec 26;11(1):e10. doi: 10.1192/bjo.2024.802 (PMC11733493; doi:10.1192/bjo.2024.802)
Supplement: Onono et al. supplementary material 3 — Onono et al. supplementary material [file S2056472424008020sup003.docx]

**COREQ – Consolidated criteria for reporting qualitative research - Checklist**

Tong A. Sainsbury P. Craig J. Consolidated criteria for reporting qualitative research (COREQ): a 32-item checklist for interviews and focus groups. International Journal for Quality in Health Care. 19(6): 349-357

| **Item** | **Response** | **Location in manuscript (section, page number)** |
| --- | --- | --- |
| ***Domain 1: Research team and reflexivity*** | | |
| *Personal Characteristics* | | |
| 1. Interviewer/ facilitator | Two trained female trilingual (English, Dholuo, and Kiswahili) qualitative interviewers, underwent rigorous training to conduct in-depth interviews with adolescents and caregivers. Both interviewers held degrees in social science and public health. | Methods, page 5 |
| 2. Credentials | MAO holds MBChB, MSc and PHD; LS holds an MPH; GO and PW both hold a MPH; EAF & AC hold a PhD, MPH; EB holds MBChB, M.Med, MPH, PhD , PGD, MBE, CIP; CG holds an MD,MPH; and SW holds an MD, MPH and MA | Author details, page 11 (Information on credentials is not available in the manuscript, only the affiliations) |
| 3. Occupation | MO is a medical doctor and Global Health Research Scientist, LS is a research data analyst at the department of medicine at UCSF. EF is a professor and the director of global health initiatives at USC. GO is a research study coordinator based at the research care and training program in KEMRI. PW is a research study coordinator based at the research care and training program in KEMRI. AC is an associate professor of medicine at UCSF. EB is Chief Research Officer & Co-Director of Research Care Training Program; CG is a professor in the department of Ob/Gyn, Reproductive Sciences and co-Director of the University of California Global Health Institute (UCGHI); SW is a professor of medicine at UCSF | Information on author occupation is not available in the manuscript, only affiliations are available |
| 4. Gender | MO, LS, GO, PW, AC, EB & SW are female; EF, CG are male |  |
| 5. Experience and training | All authors are technically and methodologically experienced researchers in Kenyan settings | Author contributions, page 11 |
| *Relationship with participants* | | |
| 6. Relationship established | Some participants might have had previous working relationship with MO, EB, GO and PW through interactions in technical working groups and interactions in the parent study | Methods, pages 3-4 |
| 7. Participant knowledge of the interviewer | Participants were informed about the reasons for the research via the invitation to participate and the consent form. | Methods: Recruitment and Consent, pages 4 |
| 8. Interviewer characteristics | No interviewer-related biases identified. |  |
| ***Domain 2: Study design*** | | |
| *Theoretical framework* | | |
| 9. Methodological orientation and theory | Thematic analysis- data was analysed using framework analysis with interpretive descriptions. The choices of thematic headings were guided by core concepts emerging out of the data using and by theoretical concepts from the design process which were predominantly aligned to the Family Stress Model | Methods: Data Analysis, page 5 |
| *Participant Selection* | | |
| 10.Sampling | Participants were purposively recruited during the end-line data collection phase of the parent study. Our Purposive sampling strategy was guided by the following variables: Among caregivers we considered the gender, age, education, marital status household size, and the study arm. Among adolescents we considered the age, education level and the study arm | Methods: Recruitment and Consenting, page 4 |
| 11. Method of approach | The current paper is a sub study of a larger matched-pair cluster, randomized controlled trial. Participants were approached during their scheduled study visits with information on the current study and asked if they would like to participate | Methods: Description of study, page 3-4 |
| 12. Sample size | Thirty-four (34) adolescents and their caregivers participated in the study | Methods: Recruitment and Consenting, page 4 |
| 13. Non-participation | All eligible and approached individuals agreed to participate in the study. | N/A |
| *Setting* | | |
| 14. Setting of data collection | Interviews were conducted in a private location of the participant’s choosing | Method: Data Collection, page 4 & 5 |
| 15. Presence of non-participants | No | N/A |
| 16.Description of sample | Adolescent girls aged 13-19 years and their primary caregivers. Adolescents aged <18 years provided assent to participate in the study  All participants provided their consent to participate in the study. | Methods: Recruitment and Consenting, page 4 |
| *Data collection* | | |
| 17. Interview guide | The semi-structured interview guides were developed in three main languages common in the study setting. The guide was developed based on existing literature and the study objectives. Topics covered in the interviews are: family composition and setup, dynamics within the family, family economic situation and, meals in a day.  The interview guide was reviewed and approved by appropriate ethics review boards. | Methods: Data collection, page 4 |
| 18. Repeat interviews | N/A | N/A |
| 19. Audio/ visual recording | All interviews were audio recorded. Audio-recordings were transcribed, translated and coded using Dedoose software. Interviews were digitally recorded and transcribed verbatim by professional transcriptionists, excluding any identifying information. All files were password-protected and stored in a secure location. | Methods: Data analysis, page 4-5 |
| 20. Field notes | Interviewers took field noted during the interviews used to enrich the transcriptions of the interviews | N/A |
| 21. Duration | Each interview lasted approximately 1 -1.5 hours. | Methods: Data Collection, page 4&5 |
| 22. Data saturation | Data saturation was discussed, and it was concluded that the data saturation was reached within the interviews conducted | Methods: Recruitment and consenting, page 4&5 |
| 23. Transcripts returned | N/A | N/A |
| ***Domain 3: Analysis and findings*** | | |
| *Data analysis* | | |
| 24. Number of data coders | A primary coder coded all transcripts in Dedoose then double coding led by a Kenyan investigator occurred with discrepancies discussed and resolved by all authors consensus | Methods: Data Analysis, page 4-5 |
| 25. Description of coding tree | The choices of thematic headings were guided by both the core concepts emerging out of the data and by theoretical concepts from the design process predominantly aligned to the Family Stress Model. Specific topics were designated as core categories; axial coding and constant comparison approach explored the relationships between the discussion of sensitive data and contextual situation | Methods: Data analysis, page 4-5 |
| 26. Deviation of themes | Specific topics were designated as core categories; axial coding and constant comparison approach explored the relationships between the discussion of sensitive data and contextual situation | Methods: Data analysis, page 5 |
| 27. Software | Dedoose qualitative software program was utilized. | Methods: Data analysis, page 5 |
| 28. Participant checking | Findings are not yet disseminated |  |
| *Reporting* |  |  |
| 29. Quotations presented | Participant quotations are provided to illustrate the themes and attributed to participants. All names accompanying quotations below are pseudonyms | Results, pages 6-9 |
| 30. Data and findings consistent | Yes | Discussion, pages 9-10 |
| 31. Clarity of major themes | Three major themes are clearly presented with rich narratives | Results, pages 6-9 |
| 32. Clarity of minor themes | Additional minor themes were in line with the framework analysis | Results, pages 6-9 |
